# A comparative analysis of compression bearing capacity in recycled concrete brick walls and composite walls incorporating coal-ash blocks

**DOI:** 10.1038/s41598-023-48369-x

**Published:** 2023-11-28

**Authors:** Jianhua Li, Xueyong Xu, Xiaoqin Liu

**Affiliations:** 1 Huzhou Vocational and Technical College, Huzhou, 313000 China; 2 Huzhou Key Laboratory of Green Building Technology, Huzhou, 313000 China

**Keywords:** Engineering, Materials science

## Abstract

In the face of the problem of waste disposal in the demolition of concrete structures, a composite wall composed of recycled concrete bricks and fly ash blocks was proposed, and based on the previous thermal performance research, its axial compression performance were further studied. Four types of walls were designed and constructed: (1) clay brick masonry (CBM), (2) recycled concrete brick masonry (RBM), (3) bilateral clay bricks masonry with coal-ash blocks sandwich insulation wall (CFCM), and (4) bilateral recycled concrete bricks masonry with coal-ash blocks sandwich insulation wall (RFRM). The test results showed that recycled concrete brick masonry exhibited a higher bearing capacity than clay brick masonry. The ultimate load of RBM was 15% higher than that of CBM. Moreover, the ultimate load of CFCM was 21% higher than that of CBM. Following the addition of sandwich coal-ash blocks in RBM, its ultimate load increased by over 42% than that of CBM. Following the addition of coal-ash blocks sandwich in both clay and recycled concrete bricks masonry, both the bearing capacity and strain exhibited improvement, the yielding load and compressive strength of them increased. Thus, it could be concluded that coal-ash blocks improved its bearing capacity. Based on the analysis of the axial compression tests, a theoretical computational model was developed and a computational expression to explain the compressive bearing capacity of a two-sided brick with coal-ash blocks sandwich insulation wall. Comparisons between the test ultimate loads (FT) and the estimated ultimate loads (FE) confirmed the accuracy of the theoretical calculation model for the compressive bearing capacity. Thus, theoretical computational models are highly recommended for the design of two-sided bricks with insulating walls constructed from coal-ash blocks being sandwiched together. This study provides a theoretical basis for the engineering application of recycled concrete brick wall and fly ash block composite wall.

## Introduction

With the continued significant improvements in the living conditions of people, urbanisation and new rural construction have also been gradually expanding in China^1^. Moreover, research on the new building materials and structural forms has been on the rise^2,3^. Khatib et al.^4^ studied the performance of reinforced concrete beams containing waste plastic straw fiber under three-point bending test. Some scholars have discussed the re-application of municipal solid waste incineration bottom ash in reinforced concrete beams^5^. Some scholars have studied the method of using waste glass sand to partially replace fine aggregate to produce low-cost, high-strength concrete^6^. The production of traditional wall materials has been proven to consume significant amounts of energy and contaminate the environment^7^. This has motivated the development of new types of wall materials using recycled materials or new forms of structures^8,9^. The considerable amounts of construction and demolition waste produced during urbanisation and new rural construction has resulted in a significant pressure on the environment^10,11^. Clay bricks, which are regarded as environmentally unfriendly building materials, have been regulated in terms of production according to the state plan in China since 2005^12^. Recycled concrete bricks, which are primarily manufactured by adopting coarse aggregates from construction waste, as well as fine aggregates and cement, which can replace clay bricks, are being promoted. This strategy can significantly reduce the amount of waste at landfills^13,14^. It has been shown that recycled concrete bricks can be used in low-rise buildings in rural areas of China^15,16^. Moreover, coal-ash blocks can also be conveniently used for construction, as they facilitate efficient use and offer the advantage of high performance under low bulk density and insulation conditions^17^. Consequently, as a low-cost filling material, coal-ash blocks have been widely employed in filler walls of multi-story masonry and reinforced concrete structures^18^. In China, coal-fired power generation produces a lot of fly ash, which not only pollutes the environment, but also takes up land when piled up. The fly ash block made of waste fly ash has the advantages of light weight, good heat preservation and so on. It is an ideal wall material^19–22^.

It is the fundamental measure to reduce the energy consumption of residential buildings to improve the energy efficiency of heating and air-conditioning by improving the thermal performance of the building envelope. The reuse of recyclable materials is also an important means to achieve the goal of energy conservation. In rural areas of China, seismic research and energy conservation research of buildings were mostly carried out separately in the past, and many houses were renovated for energy conservation after completion, resulting in waste of resources and poor integrity. With the rapid development of rural construction, it is urgent to develop new low-cost, eco-friendly and energy-saving earthquake-resistant integrated residential structure^23,24^.

To solve these problems, the author proposes Bilateral bricks with the structural form of sandwich insulation constructed using coal-ash blocks. The thermal performance of the new structure was studied and analyzed^25^. However, the compression performance of bilateral bricks with a sandwich insulation wall constructed using coal-ash blocks requires further validation. Therefore, the axial force performance of the new structure is studied in this paper.

## Property tests on axial compressive

### Design and construction of specimens

**Figure 1:**
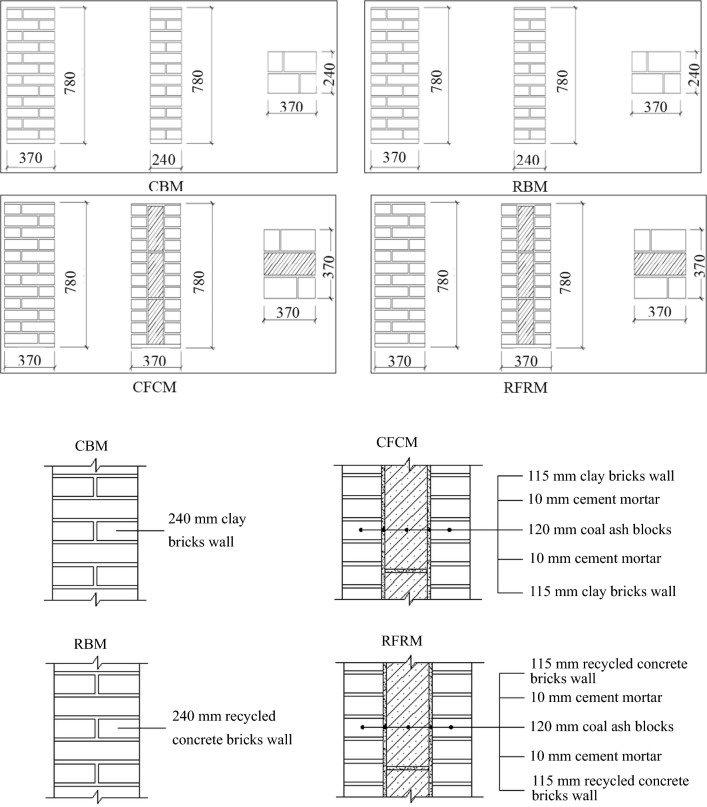
Size, thickness and structural form of wall specimen.

**Figure 2:**
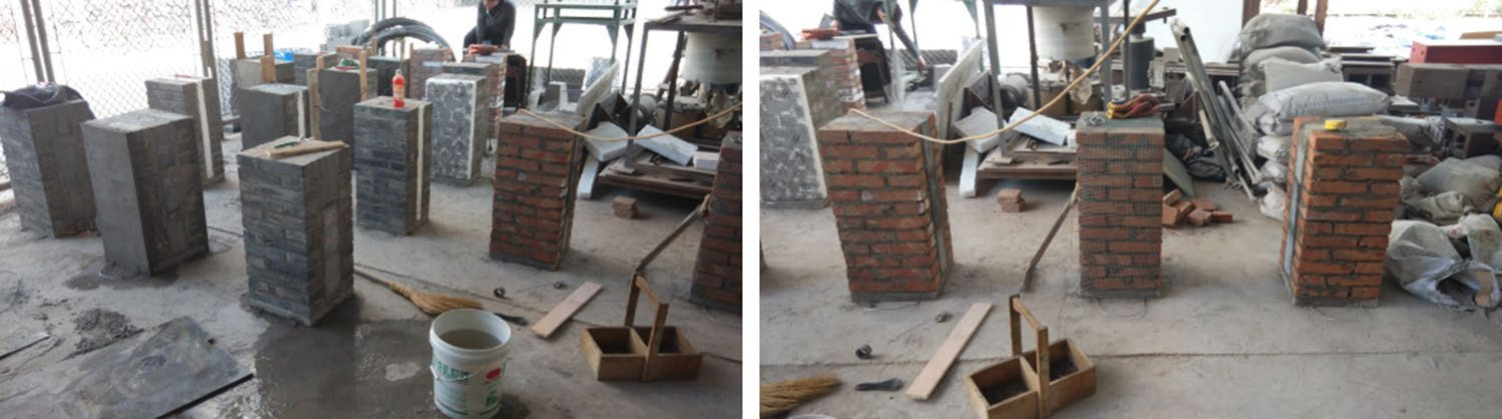
Construction process of the specimens.

Both the design and construction of specimens were based on the standard methods for basic mechanical testing of masonry (National standard of GB/T 50129-2011)^26^. Therefore, four types of specimens of the same size were tailored for the tests with width and length of 370 and 720 mm, respectively. The wire mesh layer of concrete steel with a thickness of 30 mm was set both at the top and bottom of each specimen to avoid local compression damage. In general, the thickness and structural forms of the four types were: (1) clay brick masonry (CBM) with a thickness of 240 mm; (2) recycled concrete brick masonry (RBM) with a thickness of 240 mm; (3) coal-ash blocks sandwich with a thickness of 120 mm with each bilateral clay brick (CFCM) measuring 115 mm in thickness; and (4) coal-ash blocks sandwich with a thickness of 120 mm with each bilateral recycled concrete brick (RFRM) measuring 115 mm in thickness (Fig. 1). Further, masonry mortar with a thickness of 10 mm was established for both CFCM and RFRM. In addition, a Z-shape tie bar was set at every 240 mm along the height direction. Moreover, four specimens of each type were constructed on flat ground using masonry mortar, coal ash blocks, and wire mesh layer of concrete steel with designed compressive strengths of 7.5 MPa (M7.5), 2.5 Mpa (MU2.5), and 40 Mpa (C40), respectively. The construction process of the specimens was (Fig. 2) as follows. First, 30 mm thick wire mesh layers of concrete steel were placed and left to set. Subsequently, all the wall specimens were constructed using the same mason and 30 mm thick wire mesh layers of concrete steel were set at the top of each specimen. Finally, the compression tests were conducted after 28 days.

### Performance index of materials

**Figure 3:**
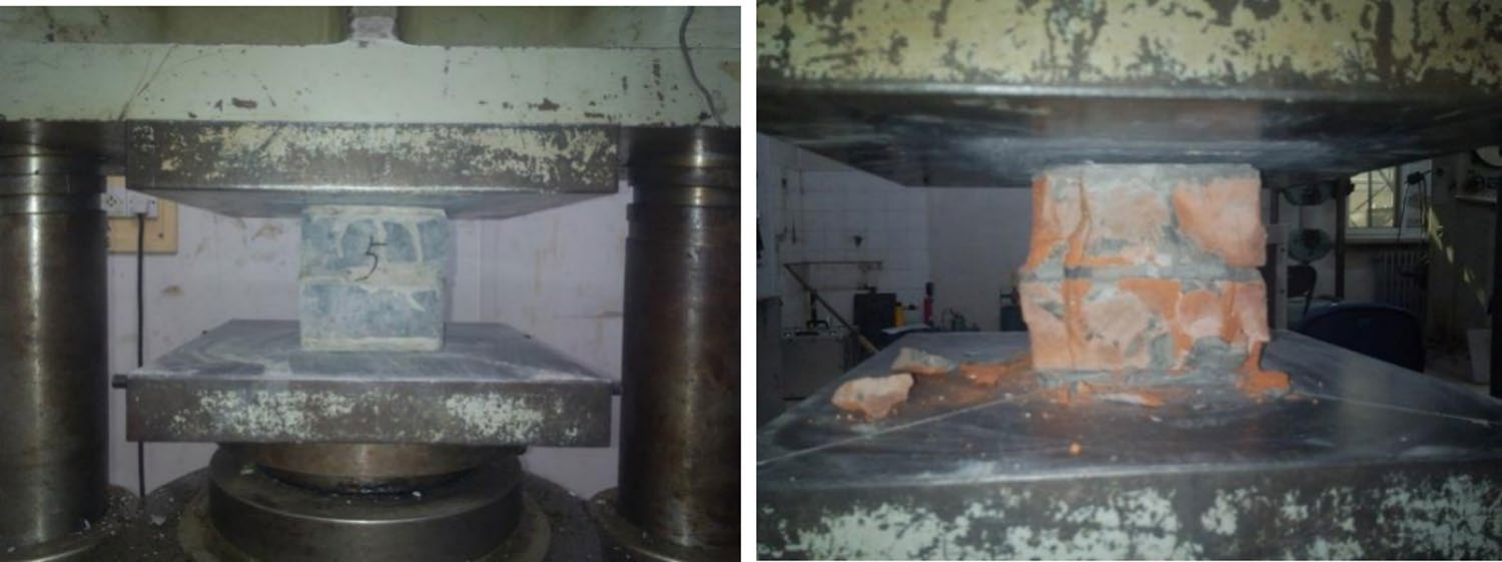
Compressive strength test of brick.

The specimen designs and compressive strength tests for clay bricks, recycled concrete bricks, coal ash block cubes, and prism were conducted based on the test methods for wall bricks (National standard of GB/T 2542-2012)^27^ and autoclaved aerated concrete (National standard of GB/T 11969-2008)^28^ (Fig. 3). The compression strength for clay bricks, recycled concrete bricks, coal ash blocks, and masonry mortar cubes were obtained as 11.05, 14.85, 2.35, and 8.37 Mpa, respectively.

### Arrangement of the monitoring point

**Figure 4:**
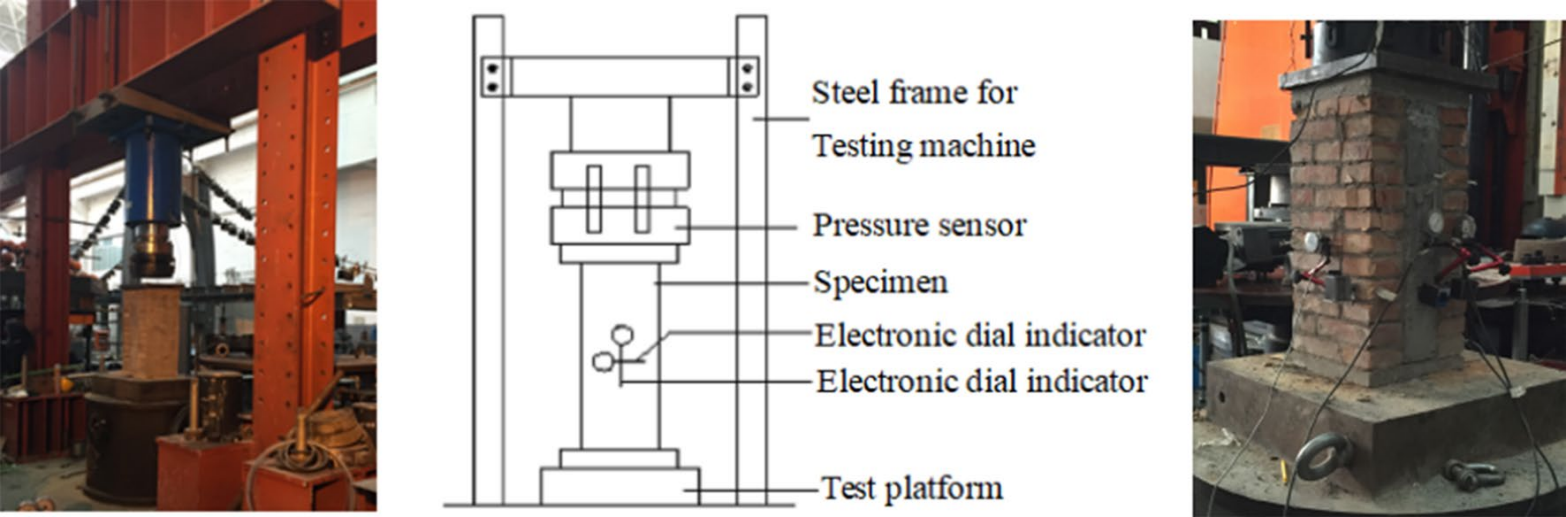
Testing apparatus.

Under the vertical uniaxial loading tests, the load-strain curve was obtained to analyse the failure progression and damage mechanism of the specimens when performing the wall compression tests. By employing electronic dial indicators mounted on the two ends of the specimens, relevant measurements were conducted for the axial compression (in the height direction) of the four structure types. In addition, the displacements (in the thickness direction) of the bilateral bricks with sandwich walls constructed using coal-ash blocks (CFCM and RFRM) were measured. Moreover, with a design range of 240 mm (Fig. 4), the measurement points for axial compression along both the height and thickness directions were set at the middle of the third point based on the standard method of the basic mechanical property test for masonry (GB/T50129-2011). The measurement points of the lateral deformation (in the width direction) were established at the horizontal centre line with a distance of 80 mm from both the left and right edges (Fig. 4). In addition, a 40 mm thick steel plate was placed on the top of the specimen, whose centre was calibrated. Furthermore, the gap existing between the steel board and the specimen was filled with fine sand to ensure uniform compression of the specimen.

### Design of loading scheme and testing procedures

Both the trisection lines and vertical centrelines along the height direction located at the two front sides and flanks of the specimen were drawn using a marking pen as per the standard method applicable for basic mechanical property test for masonry (National standard of GB/T50129-2011). In addition, the widths and thicknesses at the heights of 1/4, 1/2, and 3/4 of each specimen were measured and the average was calculated (accuracy of 1 mm). Subsequently, before the specimen was hoisted and carefully placed on the platform, with the centreline coinciding with the axis of the apparatus employed for tests, the bottom of the specimen was cleaned.

Thereafter, the sensitivity of the instruments and the soundness of the installation were examined using 5% of the estimated failure load prior to conducting the loading test. Subsequently, a repeated preloading (3–5 times) was employed with 5–20% of the estimated failure load. For the two sides, the relative errors of the axial deformation must be less than 10%; otherwise, the position of the specimen should be readjusted or levelled up.

In the load tests, the hierarchical load mode was adopted, with 10% of the estimated failure load applied continuously for 1–1.5 min. Thereafter, 10% of the estimated failure load was increased each time, and the load was maintained for 1–2 min by adjusting the amount of oil pumped into the test machine. Moreover, the entire testing process was observed during the continuous constant loads. In addition, the amount of oil pumped into the testing machine was properly reduced to decrease the strain rate to determine the failure state of the specimen during the rapid expansion of the cracks of the specimen and when the pointer of the dynamometer travelled back. The peak load value obtained from the dynamometer was considered as the failure load value for the specimen.

## Test results and analysis

### Test results

The CBM of the specimen went through three stages from the beginning of compression to failure: (1) from the beginning of compression to the initial crack. When the load is small, no damage phenomenon can be seen on the surface of the specimen. The load–displacement data curve collected by the acquisition system is approximately linear, and the specimen is in the elastic stage. When continued loading to 232 kN, vertical micro-cracks appeared in the middle brick of the specimen. (2) As the load continues to increase, cracks continue to develop along the longitudinal, the number of cracked bricks increases, and the cracks continue to develop and extend to converge. When the load reaches 300 kN, the test pieces appear through cracks, some bricks are crushed, and the skin is peeled off. (3) When the load reaches 383 kN, the load drops sharply, the axial displacement increases rapidly, the specimen is damaged, and the bearing capacity reaches the limit state.

**Figure 5:**
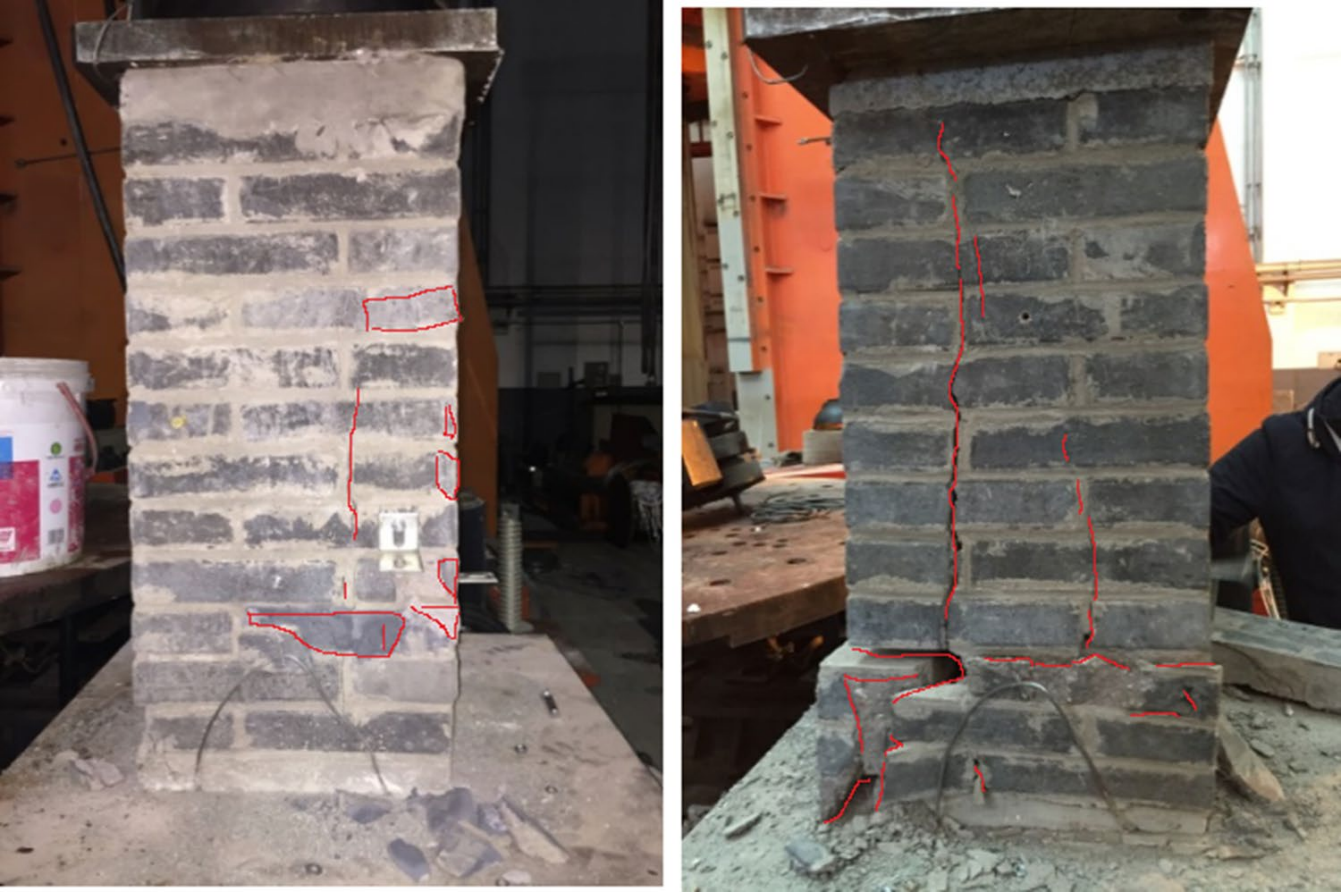
Failure patterns of RBM.

The process from initial compression to failure of specimen RBM is roughly the same as that of specimen CBM, with slightly different failure forms, such as small oblique cracks in the top brick and then small cracks in individual bricks at the bottom. With the development and extension of cracks, along with the sound of brick splitting, continuous load is applied, vertical through cracks appear, and some bricks begin to spalling. When the bearing capacity reaches the limit state, the microcracks in the test piece are less than those in the test piece CBM, and the number of spalling bricks on the surface is also less than that in the test piece CBM. The failure pattern of the test piece RBM is shown in Fig. 5.

**Figure 6:**
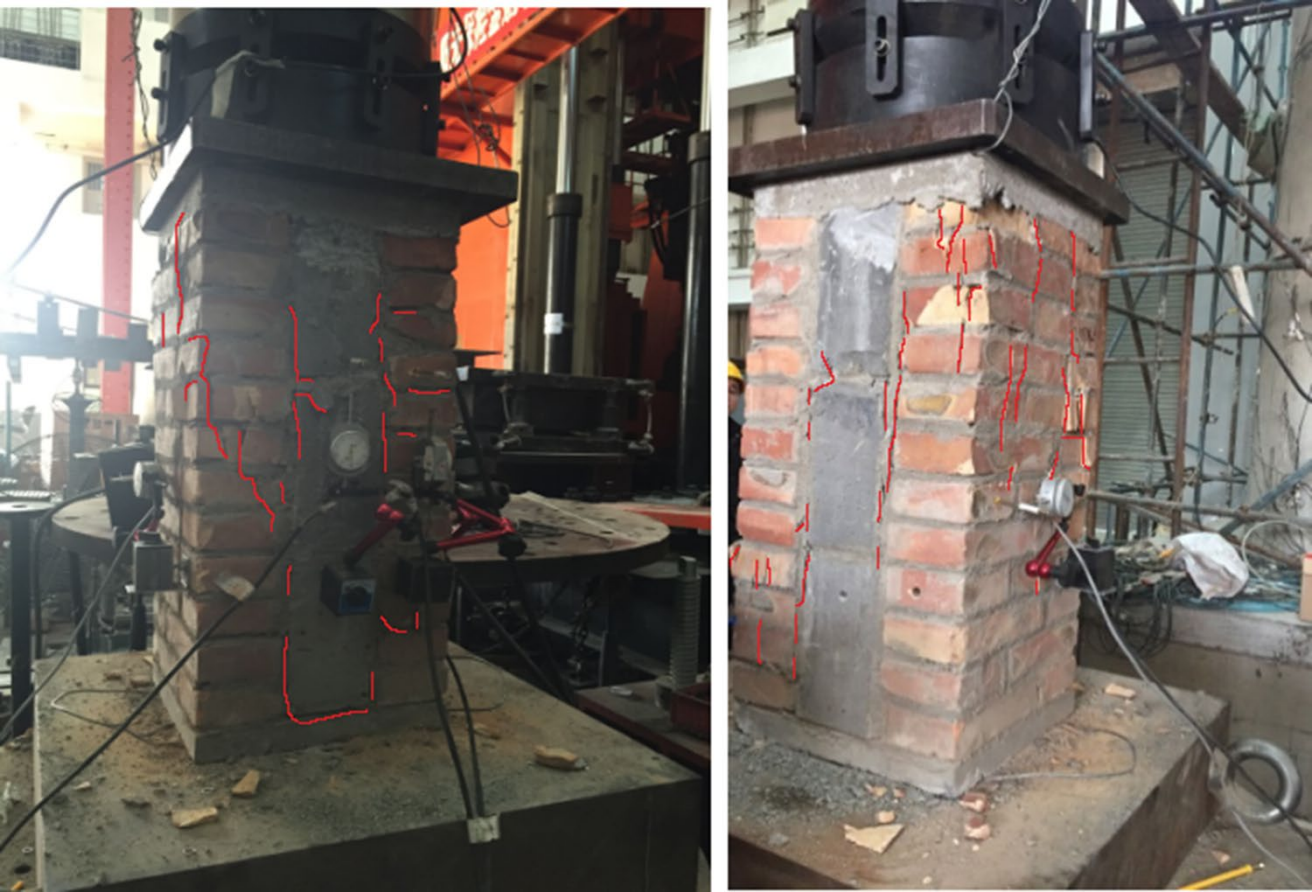
Failure patterns of CFCM.

The failure phenomena of the specimen CFCM from compression to failure are as follows: (1) when the load is small, the load–displacement curve changes linearly, and the specimen is in the elastic stage. When the specimen is loaded to 139 kN, the adhesive mortar between the clay brick and the fly ash block has a slight crack; (2) with the increase of load, the width of bonding mortar cracks between fly ash blocks and clay bricks increases slowly and extends along the length direction of bonding layer. Small oblique cracks appear and increase in front clay bricks, accompanied by the sound of brick splitting; (3) as the load continues to be applied, the vertical cracks of the front clay bricks extend, and cracks also appear in the side bricks. Some bricks begin to flake off in small pieces, and the clay bricks are divided into many small unit blocks by cracks. The load increases slowly, and the fly ash blocks begin to crack and flake and break, and the axial deformation increases rapidly, resulting in the final failure of the specimen, as shown in Fig. 6.

**Figure 7:**
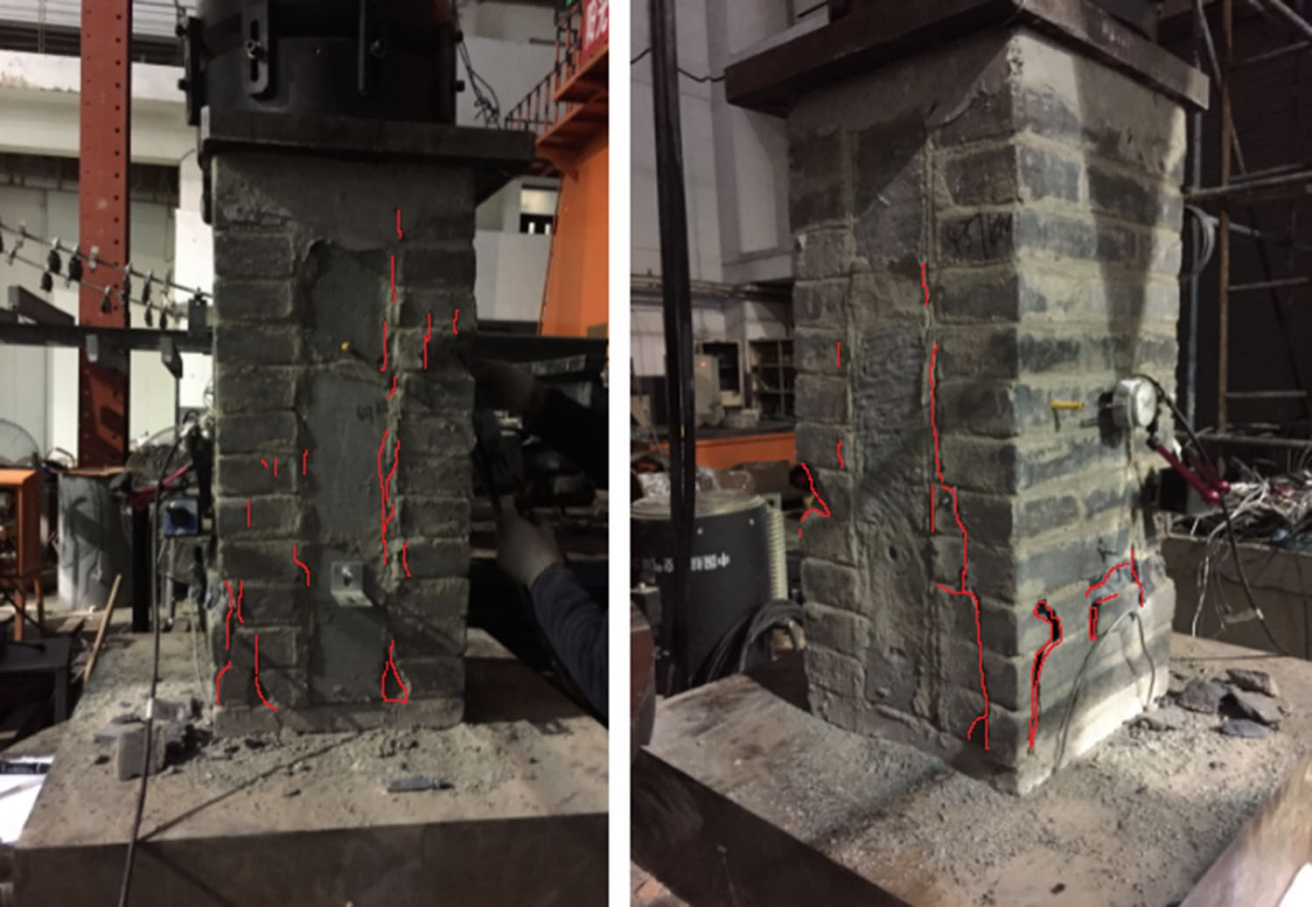
Failure patterns of RFRM.

The phenomena of RFRM from compression to failure of the specimen are as follows: (1) when the load is small, the load–displacement curve changes linearly, and the specimen is in the elastic stage. When the load reaches 121 kN, the adhesive mortar between the recycled concrete block and the fly ash block has a slight crack; (2) as the load increases, the bond mortar cracks between the fly ash block and the recycled concrete block extend, and the recycled concrete block at the front and bottom of the specimen appear small oblique cracks and gradually extend, accompanied by the sound of brick splitting; (3) as the load continues to be applied, the vertical cracks on the front side are extended, and cracks appear along the cement mortar bonding layer on the side. As a result, some bricks begin to flake off in small pieces. As the peeling area increases, the specimens lose their bearing capacity. The failure morphology of the specimen is shown in Fig. 7.

### Comparison of axial load-strain relationship for specimens of different types

**Figure 8:**
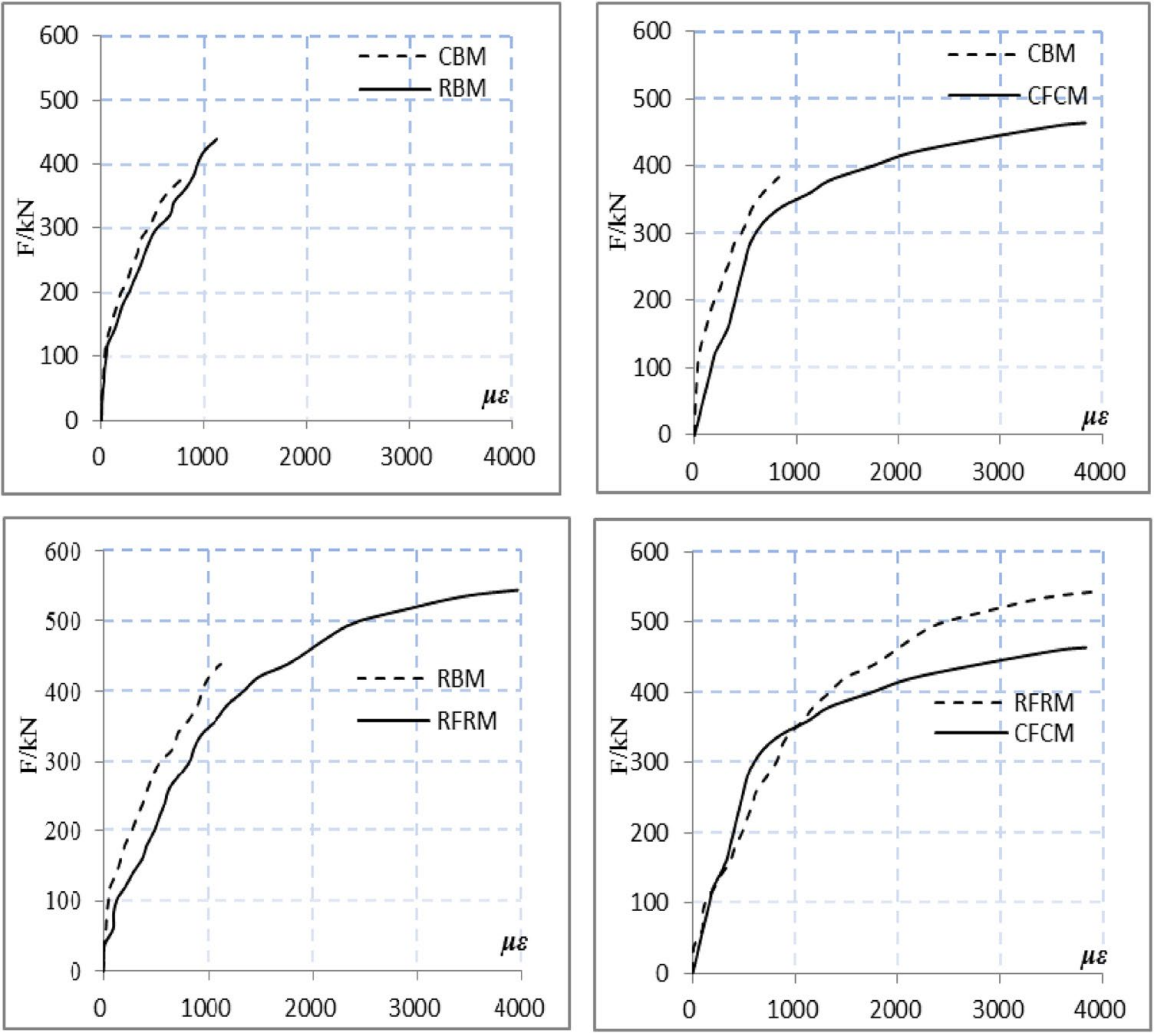
Comparison of axial load-strain relationship for specimens of different types.

The axial load–strain curve obtained for specimens of different types are shown in Fig. 8. The findings indicated that although the carrying capacity of RFRM was marginally superior than that of CFCM, both experienced brittle failure with the similar load–strain curves. Moreover, they exhibited the same progression from compression to final destruction. The comparisons between CFCM and CBM revealed that both the bearing and strain capacities improved following the addition of coal-ash blocks sandwich. Moreover, both the yield load and ductility of CFCM increased as well. In addition, the brittle failure form for CFCM was different. Whereas, the comparisons between RFCM and RBM indicated an increase in both the bearing and strain capacities following the addition of coal-ash blocks sandwich along with an improvement in the yield load and ductility. Further, the brittle failure mechanism of brick masonry was altered. A similar trend was exhibited in the relationship between the load and strain. However, compared with CFCM, RFCM exhibited a relatively higher bearing capacity and greater ductility.

**Figure 9:**
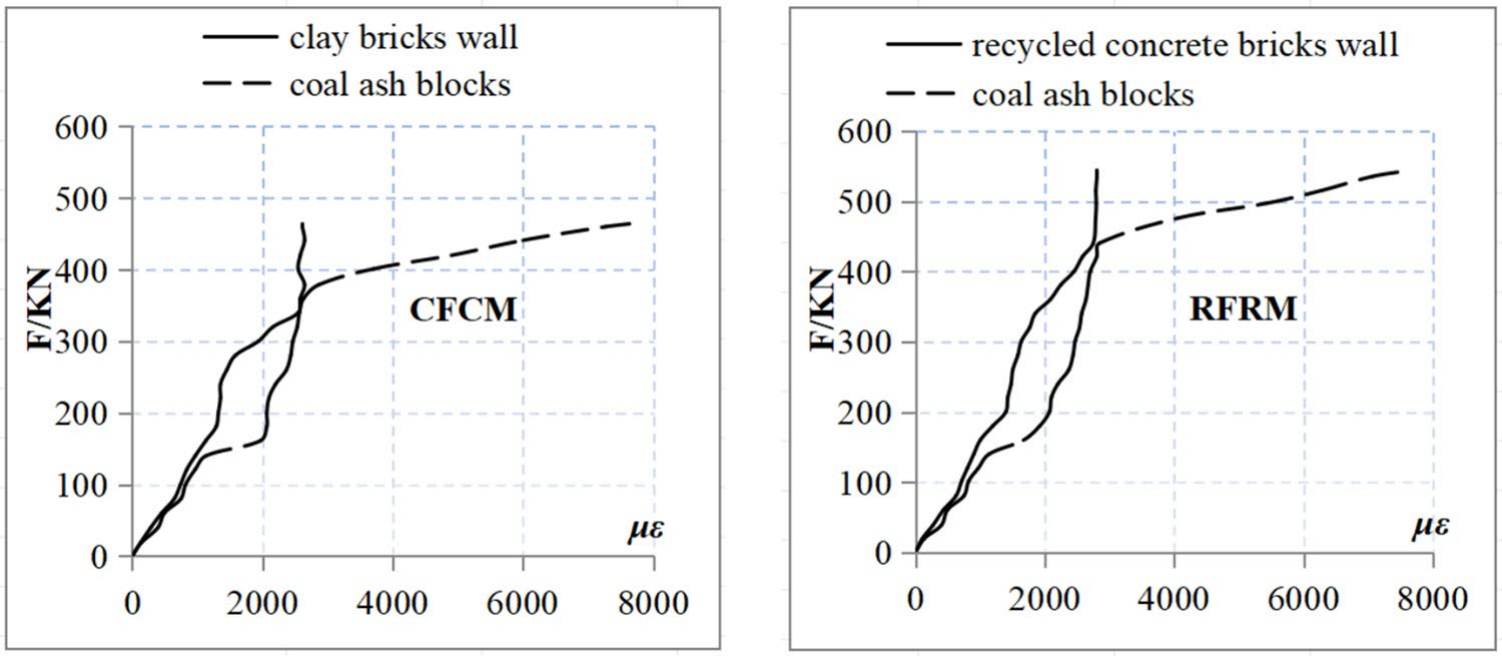
Curves of axial load-strain.

The results of stress analysis based on the load-strain curves in case of the clay and recycled concrete bricks, as well as the coal-ash blocks from CFCM and RFCM, are shown in Fig. 9. The results obtained for crack observations were as follows. (1) Prior to the appearance of micro-cracks on the mortar layer between bricks and coal-ash blocks, the loads borne by the bilateral bricks were considerably greater than that of the coal-ash blocks sandwich. The elasticity modulus obtained from the clay and recycled concrete bricks were obviously greater than that of coal-ash blocks. In addition, the mortar layer also restrained the deformation of the coal-ash blocks. (2) With an increase in the loads, mortar layer cracks and redistribution of internal force were observed between the bricks (clay and recycled concrete) and coal-ash blocks. The increase in the fly ash block strain was higher than that in the brick strain. (3) The cracks further extended and merged into the relatively wider new cracks with the continuous increase in the loads. Moreover, the strain of coal-ash blocks also increased rapidly, which indicated a dramatic increase in its bearing loads and an improvement in both the bearing capacity and ductility following the addition of coal-ash blocks.

### Analysis on bearing capacity

The cracking loads comprising the cracking load of adhesive mortar (Fs) and that of bricks (Fc), as well as the ultimate loads (Fu), and the relative value of the ultimate loads of RBM, CFCM, and RFRM to CBM (Fu_CBM_) were compared. The results are summarised in Table 1. The ultimate load of RBM was 15% higher than that of CBM, which indicated that recycled concrete bricks exhibit enormous potential in development. Moreover, the ultimate load of CFCM was 21% higher than that of CBM. However, following the addition of sandwich blocks in RBM, its ultimate load increased by over 42% than that of CBM. In addition, the cracking and ultimate loads of both the CFCM and RFRM increased. This indicated that the addition of coal-ash blocks sandwich resulted in the effects of enhanced compressive bearing capacity possessed by both CBM and RBM.

**Table 1:** Comparison between the measured cracking and ultimate loads of the specimens.

Specimens	Fs (kN)	Fc (kN)	Fu (kN)	Relative value to Fu_CBM_
CBM	–	232.22	383.53	1.00
RBM	–	277.65	439.13	1.15
CFCM	139.13	296.05	464.21	1.21
RFRM	121.35	344.33	544.32	1.42

In order to ensure the masonry quality of the specimens and avoid the test errors caused by materials and masonry reasons, all specimens are made from the same batch of materials and constructed by the same worker. Analyzing the test data, it was found that the compressive strength of recycled concrete bricks is greater than that of clay bricks, so the bearing capacity of the specimen RBM is greater than that of the specimen CBM. In addition, after the composite fly ash block is placed in the middle of the brick masonry, its cracking load is greater than that of the CBM and RBM specimens, indicating that the fly ash block also bears a portion of the axial load. As the external load continues to increase, the bonding mortar between the fly ash block and the brick body cracks, and the internal force between the brick and the fly ash block is redistributed. Due to the toughness of the fly ash block, the external load is mainly borne by the brick. As the external load continues to increase, the cracks of the brick masonry increase, the cracks were continuous and connected, and the load borne by the brick masonry decreases, while the load borne by the fly ash block increases sharply until the specimen was damaged (Fig. 9). Because fly ash blocks participate in bearing external axial loads, the failure loads of CFCM and RFRM specimens are also greater than those of CBM and RBM. In addition, because the compressive strength of recycled concrete bricks is greater than that of clay bricks, the bearing capacity of the specimen RFRM is greater than that of the specimen CFCM.

## Calculation of compressive bearing capacity for composite insulation walls

### Calculation of compressive bearing capacity

When the ultimate bearing capacity of a specimen is reached, the calculation of compressive bearing capacity of the entire specimen can be simplified to the sum of the calculation of bearing capacities of the bilateral block, fly ash blocks and bond mortar. Consequently, the ultimate compressive bearing capacity can be calculated based on the balance of vertical force shown in Eqs. (1) and (2) as follows.

1 $$ N = N_{m} + N_{f} + N_{s} $$

2 $$ N = f_{m} A_{m} + f_{f} A_{f} + f_{s} A_{f} $$

where *N*_*m*_, *N*_*f*_, and *N*_*s*_ are the compressive bearing capacities of the bilateral bricks, coal-ash blocks, and adhesive mortar, respectively, *f*_*m*_, *f*_*f*_, and *f*_*s*_ are the axial compressive stresses of the bilateral bricks, coal-ash blocks, and adhesive mortar at a given strain, respectively, and *A*_*m*_, *A*_*f*_, and *As* are the compressive areas of the bilateral bricks, coal-ash blocks, and adhesive mortar, respectively.

The compressive bearing capacity shown in the application of engineering can be calculated using Eq. (3)^29^:

3 $$ N = \psi (f_{m} A_{m} + f_{f} A_{f} + f_{s} A_{f} ) $$

where*ψ* denotes the stability coefficient of the masonry, which is specified in the code for the design of masonry structures (National standard of GB 50003-2011)^30^.

### Comparison between tested and calculated ultimate loads

Equation (3) was used to estimate the bearing capacity possessed by the bilateral bricks with insulation wall constructed using coal-ash blocks sandwich. Table 2 presents the results obtained from the tested and estimated ultimate loads. Further, the compressive strengths of both the CBM and RBM can be calculated using Eq. (4):

4 $$ f_{m} = k_{{1}} f_{{1}} ({1} + 0.0{7}f_{{2}} ) k_{{2}} $$

where *f*_*m*_ is the average compressive strength of the masonry, *f*_1_ and *f*_2_ are the average compressive strength grades for blocks and adhesive mortar, respectively, *k*_1_ is a parameter relative to the block category, *ɑ* is a parameter relative to the block and masonry category, and* k*_2_ is the correction factor of the adhesive mortar strength to compressive strength of masonry. By adopting the design standard of masonry structures, the values of *k*_*1*_,* k*_*2*_, and *ɑ* were obtained as 0.78, 0.5, and 1.0, respectively^30^.

**Table 2:** Comparison between the tested and calculated values of the ultimate load.

Specimen	Tested ultimate loads (F_T_) (kN)	Estimated ultimate loads (F_E_) (kN)	F_T_/F_E_
CBM	383.53	365.82	1.05
RBM	439.13	423.03	1.04
CFCM	464.21	470.32	0.99
RFRM	544.32	525.11	1.04
Reference^32^(RCB1)	256.00	253.05	1.01
Reference^32^(AFCB1)	298.00	324.71	0.92
Reference^32^(ARCB1)	308.00	331.34	0.93
Mean error			1.04

Regarding the brick masonry, the bearing capacity is typically expressed as follows:

5 $$ N_{m} = f_{m} A_{m} $$

In case of coal-ash blocks masonry, the determination of the compressive strength is generally conducted based on the results collected from research^31^, as well as the compressive strength of coal-ash blocks; therefore, its bearing capacity is expressed as follows:

6 $$ N_{f} \; = \;{\text{f}}_{{\text{f}}} A_{f} $$

In case of the adhesive mortar, it was assumed that it was homogeneous in the vertical direction and its bearing capacity could be directly obtained by employing its compressive strength and cross-sectional area.

The theoretical calculation model was used to calculate the four types of specimens, and the similar core column specimens in the reference^32^ were verified. The calculation results are shown in Table 2.

As can be seen from Table 2, the theoretical calculation results agree well with the test results in general, and the mean error is less than 4%. The results show that the bearing capacity calculation model proposed in this paper is suitable for double-layer masonry with fly ash block interlayer and recycled concrete brick masonry.

## Conclusions

Four types of specimens were tested to investigate their axial compressive property. The results showed that recycled concrete brick masonry exhibited a higher bearing capacity than clay brick masonry, which indicated the enormous development potential in terms of recycled concrete bricks for constructions in rural area. Moreover, following the addition of coal-ash blocks sandwich in both clay and recycled concrete bricks masonry, both the bearing capacity and strain exhibited improvement, the yielding load and tensile strength of them increased. The ultimate load of RBM was 15% higher than that of CBM. Moreover, the ultimate load of CFCM was 21% higher than that of CBM. Following the addition of sandwich blocks in RBM, its ultimate load increased by over 42% than that of CBM. In addition, the cracking and ultimate loads of both the CFCM and RFRM increased. Thus, it could be concluded that coal-ash blocks enhanced the brittle failure characteristics of brick masonry and improved its bearing capacity. The computation expression of bearing capacity for bilateral bricks with an insulation wall constructed using coal-ash blocks sandwich was subjected to a comparative analysis to test and calculate the values theoretically. The results confirmed the correctness and feasibility of the calculation model. Moreover, it can be employed for the design of similar wall types.

In the face of the problem of waste disposal in the demolition of concrete structures, based on the previous thermal performance research of the composite wall of recycled concrete brick and fly ash block, the axial compression mechanical properties of the wall are studied in this paper. According to the research, the thermal insulation and mechanical properties of recycled concrete brick wall and fly ash block composite wall are improved compared with the traditional clay brick wall, and can replace the traditional clay brick wall. For the seismic area, further research on seismic performance is needed.

## Data Availability

All data generated or analysed during this study are included in this published article.
